# Text Mining for Literature Review and Knowledge Discovery in Cancer Risk Assessment and Research

**DOI:** 10.1371/journal.pone.0033427

**Published:** 2012-04-12

**Authors:** Anna Korhonen, Diarmuid Ó Séaghdha, Ilona Silins, Lin Sun, Johan Högberg, Ulla Stenius

**Affiliations:** 1 Computer Laboratory, University of Cambridge, Cambridge, United Kingdom; 2 Institute of Environmental Medicine, Karolinska Institutet, Stockholm, Sweden; University of Illinois-Chicago, United States of America

## Abstract

Research in biomedical text mining is starting to produce technology which can make information in biomedical literature more accessible for bio-scientists. One of the current challenges is to integrate and refine this technology to support real-life scientific tasks in biomedicine, and to evaluate its usefulness in the context of such tasks. We describe CRAB – a fully integrated text mining tool designed to support chemical health risk assessment. This task is complex and time-consuming, requiring a thorough review of existing scientific data on a particular chemical. Covering human, animal, cellular and other mechanistic data from various fields of biomedicine, this is highly varied and therefore difficult to harvest from literature databases via manual means. Our tool automates the process by extracting relevant scientific data in published literature and classifying it according to multiple qualitative dimensions. Developed in close collaboration with risk assessors, the tool allows navigating the classified dataset in various ways and sharing the data with other users. We present a direct and user-based evaluation which shows that the technology integrated in the tool is highly accurate, and report a number of case studies which demonstrate how the tool can be used to support scientific discovery in cancer risk assessment and research. Our work demonstrates the usefulness of a text mining pipeline in facilitating complex research tasks in biomedicine. We discuss further development and application of our technology to other types of chemical risk assessment in the future.

## Introduction

New research in biomedicine depends on making efficient use of existing scientific knowledge – a task which bio-scientists are finding increasingly difficult. Given the double exponential growth rate of biomedical literature over recent years [1], there is now a pressing need to develop technology that can make information in published literature more accessible and useful for scientists. Such technology can be based on text mining. Drawing on techniques from natural language processing, information retrieval and data mining, text mining can automatically retrieve, extract and discover novel information even in huge collections of written text. Although it cannot yet replace humans in complex tasks, it can enable humans to identify and verify required information in literature more efficiently and uncover relevant information obscured by the volume of available information.

In recent years, biomedical text mining has increased in popularity. Techniques have been developed to assist, for example, the extraction of documents, databases, dictionaries, ontologies, summaries and specific information (e.g. interactions between proteins and genes, novel research hypotheses) from relevant literature [2]–[4]. Evaluation of such techniques has revealed promising results. However, much of the evaluation has been direct in nature and has employed pre-determined gold standards. There is now general recognition of the need to move biomedical text mining research closer to practice: to integrate technology to support real-life scientific tasks (e.g. the process of scientific discovery) and to evaluate its usefulness in the context of such tasks [3], [5].

A number of studies have responded to this need for user-centred evaluation, though the undertaking of user studies is still far from universal. Some studies have measured the degree to which semi-automation can speed up a curation or other workflow [6]–[8]. A second strand, more closely related to our work, seeks to discover new relationships between biological entities that are supported by but not made explicit in the literature [9]–[11]; for example, the existence of a known link between a disease and a gene and between the same gene and a drug might suggest a role for the drug in treating the disease. User evaluation in this context involves comparing the proposed relationships to previously suggested hypotheses and making qualitative judgements as to whether they seem to offer fruitful directions for further research. Our case studies follow the same basic template, though the task at hand, requiring synthetic analysis of full abstracts, is a more complex one than classifying relations between entity mentions.

In this paper we present a new, fully integrated text mining system designed to support the complex and highly literature-dependent task of chemical health risk assessment. This task is critical because chemicals play an important role in everyday life and their potential risk to human health must be evaluated. With thousands of chemicals introduced every year, many countries worldwide have established increasingly strict laws governing their production and use. For example, the recent European Union Registration, Evaluation, Authorisation and Restriction (REACH) legislation [12] requires that all chemicals manufactured or imported in large quantity must undergo thorough risk assessment.

The assessment of large numbers of chemicals is easier said than done. Using the currently available methodology, it takes up to two years to assess a single chemical [13]. Although the development of a completely novel system for toxicity testing may help to improve the efficiency of chemical assessment in the long term [14], there is a pressing need to improve the state of the art in the short to medium term.

Chemical risk assessment is a complex process consisting of several component stages. The first major component is typically an extensive review and analysis of the available scientific data on the chemical in question. This review focuses on any data of potential relevance – not only human data, but also animal, cellular (in vitro) and other mechanistic data [15]. The primary source for this data is scientific peer reviewed literature.

According to a recent report, risk assessors find literature gathering and analysis prohibitively time-consuming [16]. This is not surprising since the biomedical sciences which chemical risk assessment draws on (epidemiology, cell biology, and cancer research, among many others) are developing more rapidly than ever before. This development can be observed by examining the growth of MEDLINE (Medical Literature Analysis and Retrieval System Online) - the U.S. National Library of Medicine's (NLM) premier bibliographic database which is a significant literature resource employed in current chemical risk assessment. In 2005, this database included 13 million references. Today it includes over 18 million, with 2,000–4,000 references added to MEDLINE each day; in fact, the database is growing at a double-exponential rate [1]. The data for a single chemical may be found scattered across thousands of journal articles (e.g. MEDLINE includes over 30,000 articles for cadmium).

At present, risk assessors and scientists use systems such as PubMed to gather relevant literature from databases. These systems return a list of journal articles in response to keyword-based queries. However, given the wide range and complexity of scientific data used for risk assessment, the number of keywords, their synonyms and potential combinations simply exceeds what human risk assessors can reasonably memorize and handle. What is essentially needed is much more powerful technology which goes beyond keyword-based search – technology which categorizes and ranks various scientific data on the basis of their relevance, makes links between otherwise unconnected articles, and creates summaries, statistics, visualizations and novel hypotheses from the scientific literature, leaving risk assessors to explore the resulting structured data. The work reported here shares some of the goals of the Semantic MEDLINE project [17], [18] in adding a “semantic” layer of automatic processing over the keyword-based retrieval functionality of PubMed or a similar search engine. We believe that our work is distinguished from Semantic MEDLINE by our use of statistical NLP methods, by the focus on an underexplored task setting with a distinctive information need and by our focus on user-centred evaluation.

If a dedicated text mining tool was developed for chemical risk assessment it could be used to effectively identify, mine, and classify scientific data in biomedical literature as well as to discover novel patterns in classified data. Facilitating large-scale assessment of existing data, such a tool could offer the means to improve the accuracy, thoroughness and efficiency of chemical risk assessment. The tool could also be used to support scientific research in the fields on which risk assessment relies.

In Korhonen et al. [16] we took the first step towards the development of text mining technology for chemical risk assessment, focussing on cancer risk assessment. We introduced a basic taxonomy which covers the main types of scientific evidence used for determining carcinogenic properties of chemicals, and a supervised machine learning approach which can be used to classify MEDLINE abstracts to relevant taxonomy classes. The evaluation showed that the taxonomy is well-formed and that the machine learning approach is fairly accurate. Although the experiment was small in scale and no evaluation of the practical usefulness of the technology for real-life risk assessment was performed, the results were nevertheless promising.

We take this line of research considerably further and introduce CRAB – a fully integrated text mining tool aimed at supporting the entire process of literature review and knowledge discovery in cancer risk assessment. Available to end users via an online Web interface, it enables accessing PubMed, downloading scientific abstracts on chosen chemicals, and classifying them according to an extensive taxonomy using supervised machine learning technology. The tool allows navigating the classified dataset in various ways and sharing the data with other users. We present both direct and task-based evaluation of the technology integrated in the tool, along with a number of case studies which demonstrate the usefulness of the tool in supporting knowledge discovery in cancer risk assessment and research.

Our research demonstrates that a relatively ambitious text mining pipeline consisting of both retrieval and multi-classification stages can be useful for complex research tasks in biomedicine. Although currently applicable to cancer, the tool could be straightforwardly adapted to support the assessment and study of other important health risks related to chemicals (e.g. allergy, asthma, reproductive disorders, among many others).

## Methods

The following three sub-sections describe the key components of CRAB: the cancer risk assessment taxonomy, the corpus of MEDLINE abstracts annotated according to the taxonomy classes, and the classifier based on machine learning. The final sub-section presents the overall architecture of the CRAB tool along with the user interface.

### Taxonomy

At the heart of CRAB is a taxonomy developed by experts in cancer research, which specifies scientific data types of relevance for cancer risk assessment. We took the taxonomy of Korhonen et al. [16] as a starting point and extended and refined it in various ways. The resulting taxonomy includes data types mentioned in publicly available cancer risk assessment guidelines (e.g. US EPA Guidelines [15]) as well as additional, more detailed and recent data discovered during expert analysis of risk assessment literature.

**Figure 1:**
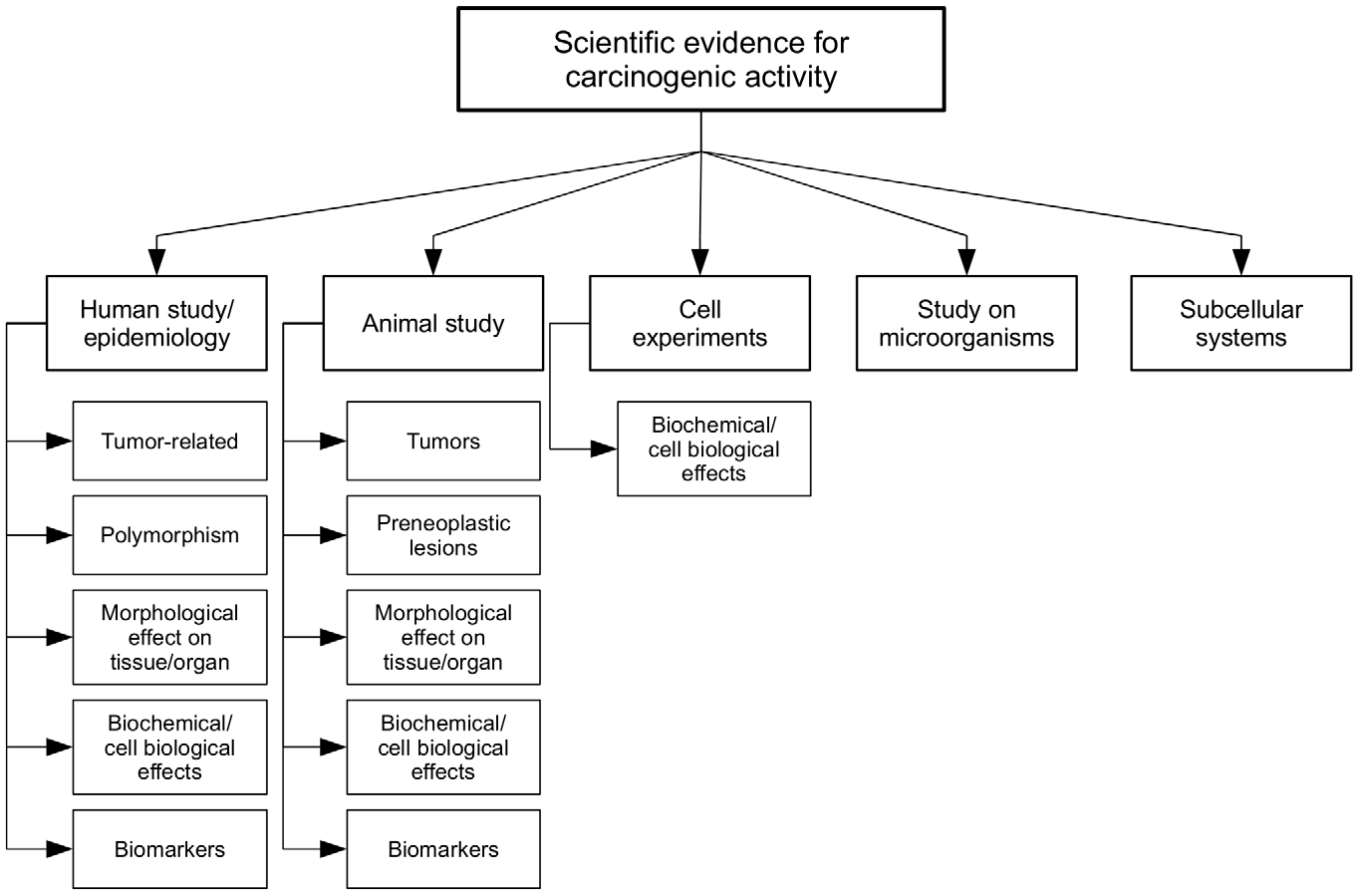
The *Scientific Evidence for Carcinogenic Activity* taxonomy branch.

The taxonomy has two main parts. The first part (shown in Figure 1) focuses on *Scientific Evidence for Carcinogenic Activity*. It has five top level classes which represent different types of scientific evidence: *Human study/Epidemiology*, *Animal study*, *Cell experiments*, *Study on micro-organisms*, and *Subcellular systems*. Some of these divide further into sub-classes; for example, *Human study* has five sub-classes including *Tumor-related* and *Polymorphism*. We adopted all of the top level classes and the majority of sub-classes proposed by Korhonen et al. [16].

**Figure 2:**
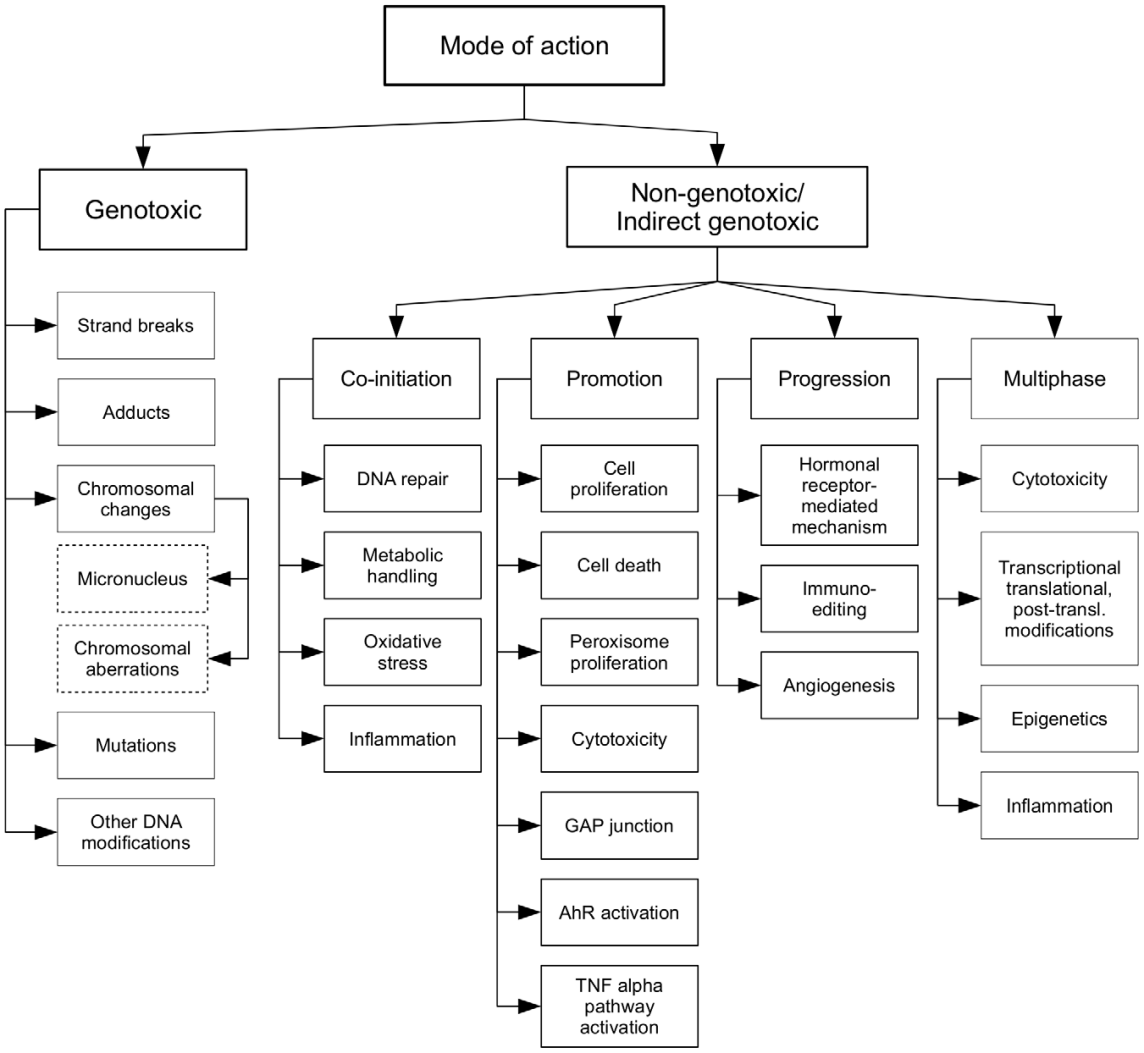
The *Mode of Action* taxonomy branch.

The second part of the taxonomy (shown in Figure 2) focuses on *Mode of Action* (MOA; i.e. the sequence of key events that result in cancer formation, e.g. mutagenesis, increased cell proliferation, and receptor activation), capturing the current understanding of different processes leading to carcinogenesis. We took the simple MOA taxonomy of Korhonen et al. [16] which distinguishes two commonly used MOA types – *Genotoxic* (i.e. a carcinogen binds to DNA) and *Non-genotoxic/indirect genotoxic* (i.e. a carcinogen does not bind to DNA) – as a starting point. We added four sub-classes under the *Non-genotoxic/indirect genotoxic* class (*Co-initiation*, *Promotion*, *Progression* and *Multiphase*), following the recently proposed MOA classification of Hattis et al. [19]. Each of these classes divides further into sub-classes according to the types of evidence that can indicate the MOA type in question. For example, *Cytotoxicity* can provide evidence for both *Promotion* and *Multiphase* non-genotoxic MOAs.

**Figure 3:**
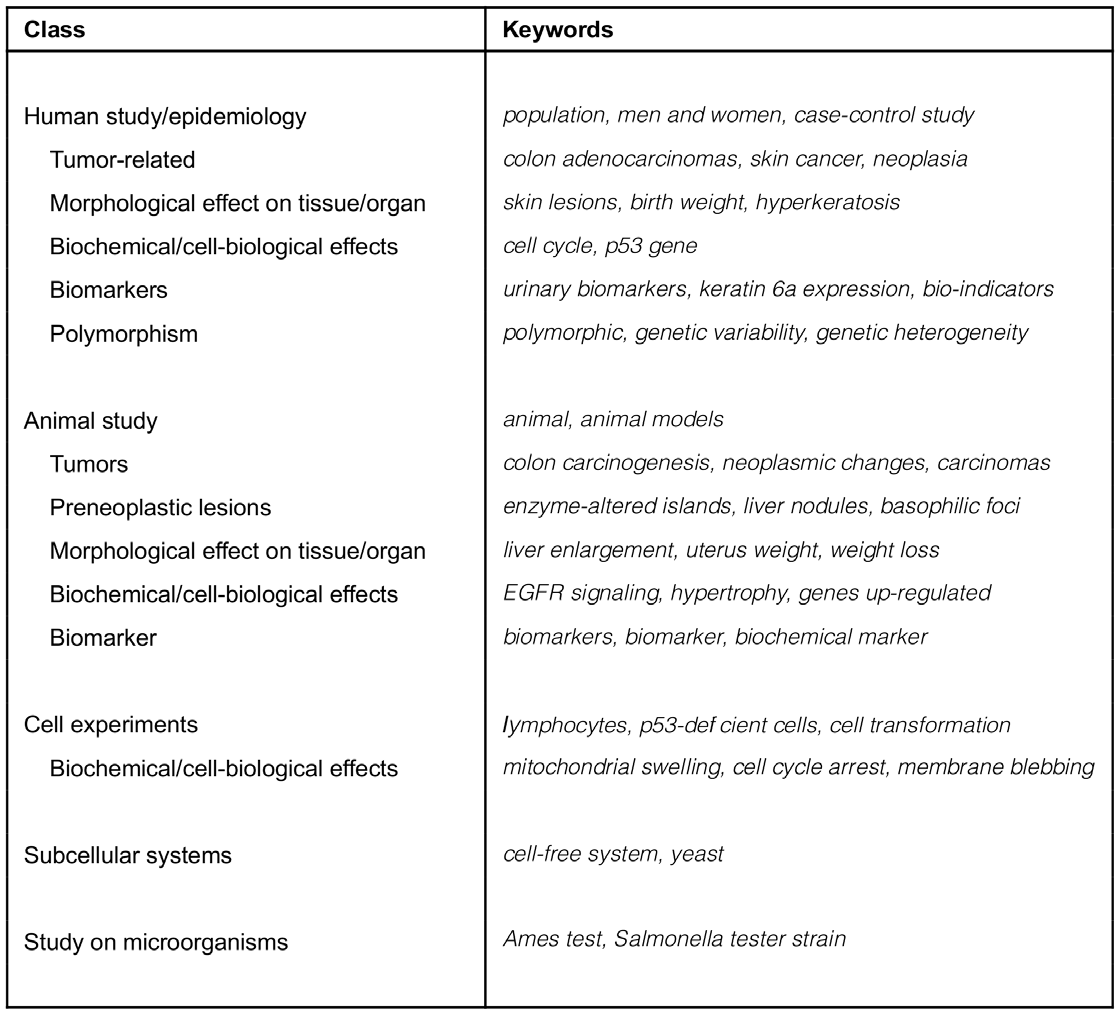
Example keywords for the *Scientific Evidence for Carcinogenic Activity* taxonomy.

**Figure 4:**
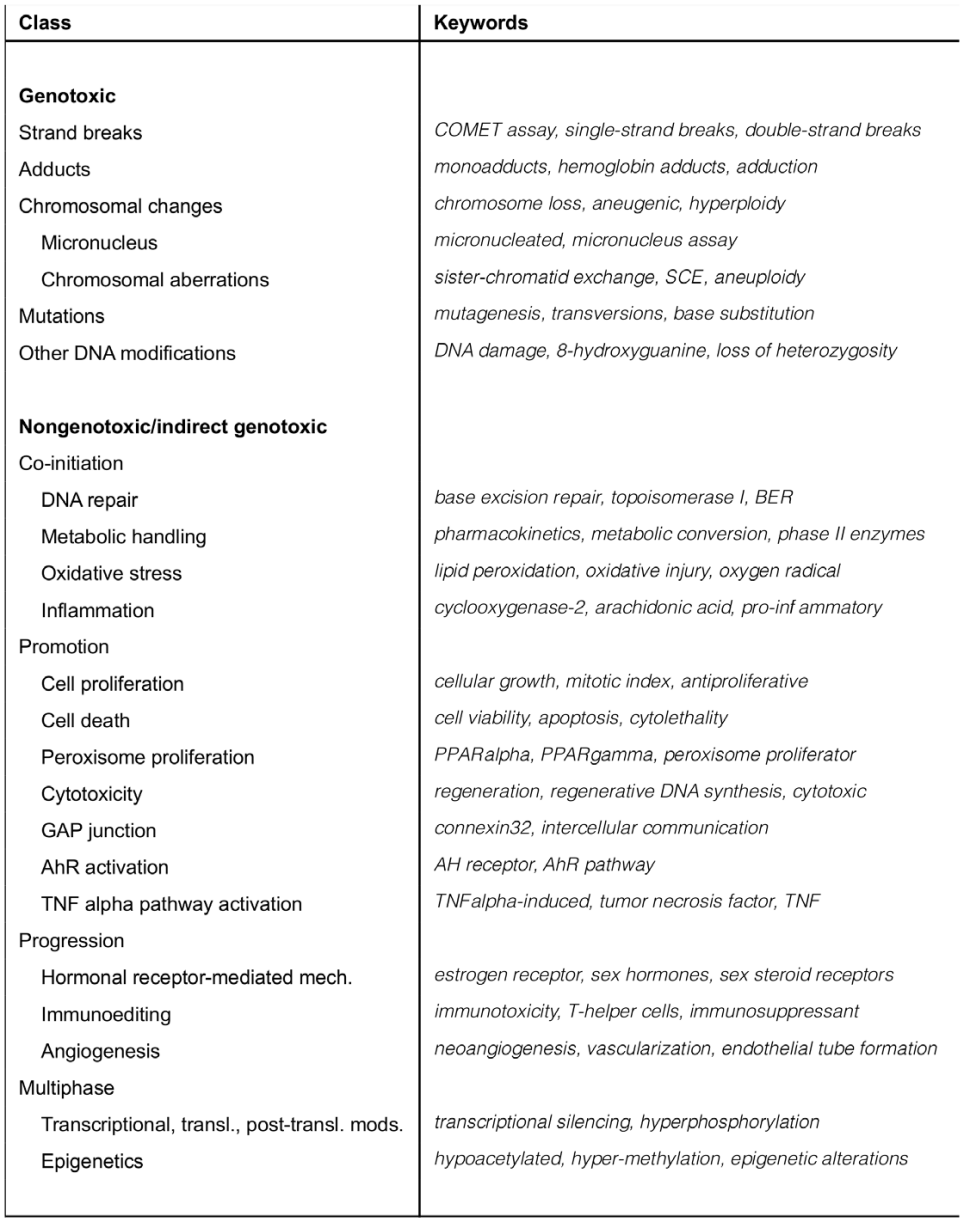
Example keywords for the *Mode of Action* taxonomy.

The resulting taxonomy contains 47 classes. Each class is associated with a number of keywords (and keyphrases) which, when found in literature, are good indicators for the presence of the type of scientific data in question (e.g. the *Cell death* class in the *MOA* part of the taxonomy includes keywords such as *apoptosis*, *DNA fragmentation*, *caspase-9*, *bcl2*, *bax*, *apoptosome*, *programmed cell death*, *Fas*, *necrotic cell death*, and *viability*). Figure 3 shows representative keywords for each class in the *Scientific Evidence for Carcinogenic Activity* taxonomy branch. Figure 4 presents example keywords for the *MOA* taxonomy branch. The keywords shown were selected from the annotated corpus described below.

Due to the rapid development of science a taxonomy like this will never be complete. However, it can be extended and updated easily by experts using our tool.

### Annotated Corpus

The CRAB classification software requires as training data a corpus (i.e. a collection) of MEDLINE abstracts that have been manually classified according to the taxonomy. The Korhonen et al. [16] corpus was created by selecting eight chemicals which are (i) well-researched using a wide range of scientific tests and which (ii) represent the two most frequently used MOAs (*genotoxic* and *non-genotoxic*): 1,3-butadiene, benzo(a)pyrene, diethylnitrosamine, styrene, chloroform, diethylstilbestrol, fumonisin B1 and phenobarbital. A set of 15 journals were then identified which are used frequently for cancer risk assessment and jointly provide a good coverage over the different types of scientific evidence relevant for the task (e.g. Cancer Research, Carcinogenesis, Environmental Health Perspectives, Mutagenesis, among others). From these journals, all the abstracts returned by PubMed for the years 1998 to 2008 which include one of the 8 chemicals were downloaded (1297 abstracts in total). Each abstract was then examined by an expert in cancer risk assessment and assigned to relevant taxonomy classes via keyword annotation. An annotation tool was developed and used in this work (see Korhonen et al. [16] for details).

The annotated dataset is available under a Creative Commons Attribution Non-Commercial license (Information S1 and S2); as far as we are aware, this is the first time that a corpus of chemical risk annotation data has been publicly available.

**Figure 5:**
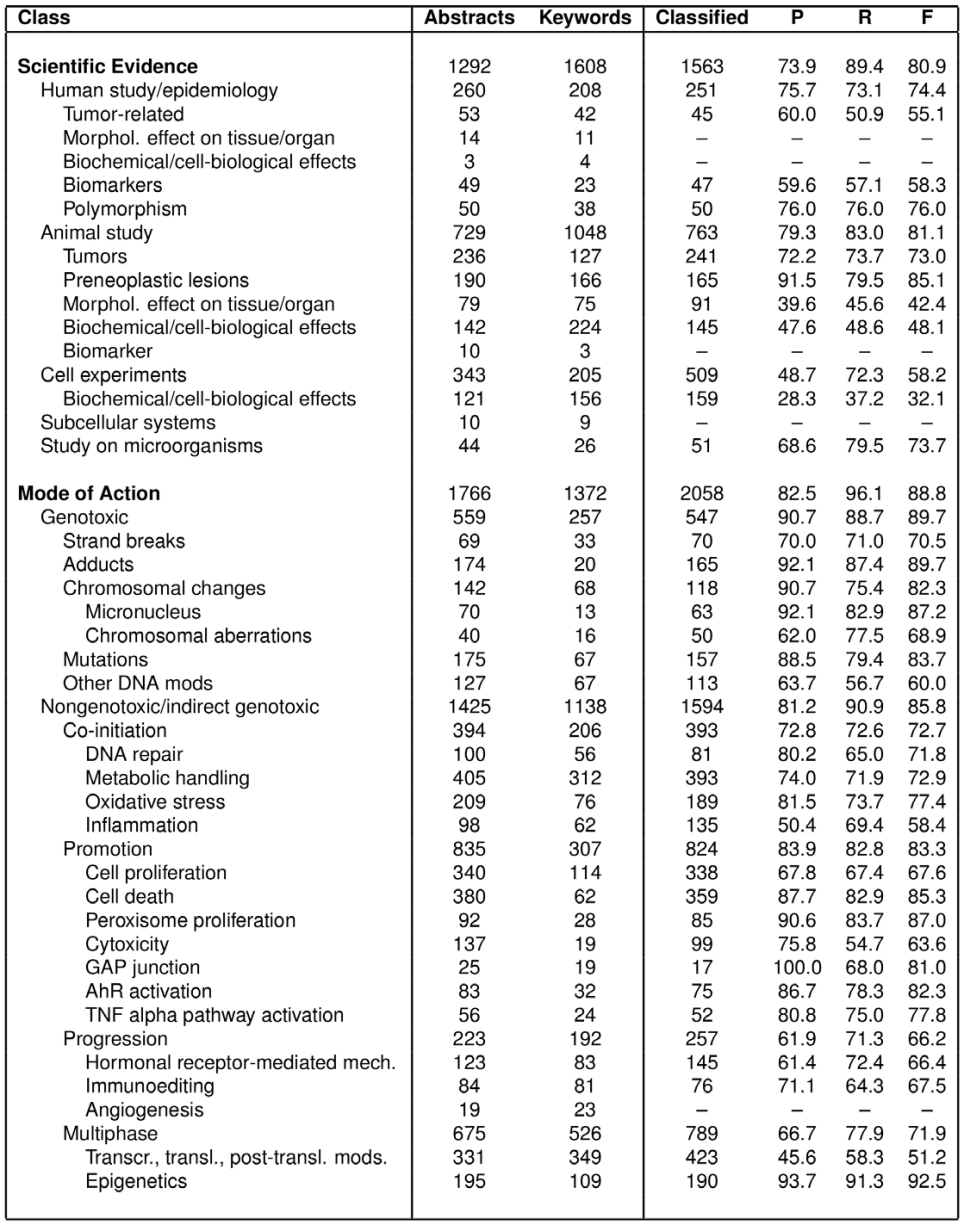
Classification results: number of abstracts and distinct keyword annotations for each label; number of abstracts classified as positive by the system; Precision, Recall and F-measure.

We re-annotated the corpus of Korhonen et al. [16] using our taxonomy and extended it considerably: we selected twelve additional chemicals (shown in Table 1) – ones that collectively represent the types of scientific evidence and MOAs covered by our extended taxonomy. Abstracts returned by a PubMed search for these chemicals (all from the years 1999–2009) were downloaded and annotated by cancer risk assessors using the annotation tool of Korhonen et al. [16]. The resulting combined corpus consists of 3078 annotated MEDLINE abstracts for 20 chemicals. The total number of abstracts and annotated keywords belonging to each taxonomy class is shown in Figure 5 (see columns 1–3). We can see that 1292 abstracts have been classified according to the *Scientific Evidence for Carcinogenic Activity* sub-taxonomy, while 1766 have been classified according to the MOA taxonomy. The number of abstracts and individual keywords associated with top level classes is high but get increasingly small as we go into the deeper levels of the taxonomy.

**Table 1:** Profiles of the new chemicals used for annotation.

Chemical	Occurrence	Effects
5-azacytidine	Used in the treatment of leukemia	DNA Methylation, cytotoxicity
Arsenic	A metalloid found in many minerals	Oxidative stress, cell death, angiogenesis
Bisphenol A	Used in the manufacture of plastics	Endocrine disruptor
Cadmium	A metal (metal ion)	DNA repair inhibition, oxidative stess
Cyclosporine	Immunosuppressant drug	Immunosuppression, apoptosis
Dichloroacetate	Used for treatment of lactic acidosis	Methylation, cell death, oxidative stress
Irinotecan	Drug used for cancer treatment	Topoisomerase inhibition, immunosuppression
Nafenopin	Drug used for blood lipid levels	Peroxisome proliferation
Okadaic acid	A marine toxin	Protein phosphatase inhibition and effects on TNF-alpha
Sulindac	An anti-inflammatory drug	Reduced inflammation
TCDD	A dioxin-like compound	AhR activation and other
Thiobenzamide	Hepatotoxin	Immunosuppression

### Classification experiments

### Classifier

The CRAB classifier assigns unseen MEDLINE abstracts to appropriate taxonomy classes using a supervised machine learning technique. The technique does not rely on pre-defined keywords, but it uses a set of linguistic document features (described below) and the associated corpus annotations (described in the above section) as training data to achieve optimal performance.

Korhonen et al. [16] used a set of Support Vector Machine (SVM) classifiers [20], one for each taxonomy class, to decide which (if any) taxonomy classes describe the content of an abstract. Since SVMs have performed well in many text mining tasks [2], [21] and since they yielded promising results in the preliminary experiments of Korhonen et al. [16] we use them also in our system. However, we introduce an improved model and additional features to obtain better performance on our task.

Similar to other well-known classifiers such as logistic regression or the perceptron, SVMs separate a training dataset into two classes by learning a decision function that corresponds to a combination of feature values and feature weights. For SVMs this function can be written as:
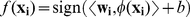
(1)where 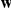 is a vector of weights learned from training data and 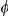 is a function that maps datapoints from the input space to a (potentially different) “feature space”. The SVM training algorithm sets the weight vector in correspondence with the *max-margin* principle, choosing the boundary that maximises the separation between classes. Often the feature space mapping 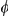 need not be computed directly as its effect can be captured via the use of a *kernel function* that compares two datapoints; this allows SVMs to learn non-linear decision boundaries while maintaining the computational efficiency of linear classification. The books [22], [23] provide comprehensive overviews of SVMs and of kernel methods in general.

One standard kernel function is the dot product or *linear kernel*, which we used in Korhonen et al. [16]:
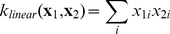
(2)An alternative kernel function, suitable for comparing probability distributions (or L1-normalised vectors), can be derived from the Jensen-Shannon divergence (JSD) [24] through a method proposed by Hein and Bousquet [25]:
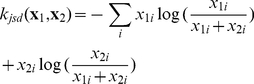
(3)Ó Séaghdha and Copestake [26] demonstrate that this *JSD kernel* yields substantially better performance than the linear kernel on a range of classification tasks in natural language processing; hence we apply it here with the expectation that it will improve the accuracy of our automatic abstract annotation.

Abstracts are input to the classification pipeline as PubMed XML, from which the content of each abstract and some associated markup are extracted. The abstract text is tokenised (split into its component word tokens) using the OpenNLP toolkit [27] and transformed into a “bag of words” feature vector that stores the number of times each word occurs in the text. A separate set of features records the words that appear in the abstract title, to capture the intuition that the title words have a privileged status in identifying the principal theme of an article. These features are augmented by the MeSH (*Medical Subject Headings*) headings provided by MEDLINE; for example, an abstract may have been given the descriptive headings *Drug Interactions* and *Enzyme Inhibitors*. The parent categories or *hypernyms* of these headings in the MeSH taxonomy are also added; for example, the hypernyms of *Enzyme Inhibitors* include *Molecular Mechanisms of Action* and *Pharmacologic Actions*. Finally, all character strings of length 7 (including sentence-internal punctuation and spaces) are extracted from the text and converted to another set of features; the proposed sequence length of 7 follows Wang et al. [28], but the use of character-based features for string comparison has a long history in bioinformatics, e.g. the *spectrum kernel* of Leslie et al. [29].

Compared with the system of Korhonen et al. [16], our system integrates the following refinements: (1) the use of the JSD kernel rather than the linear kernel; (2) the use of title word features; (3) the addition of MeSH hypernyms.

The classifier associated with each taxonomy class predicts a binary label; an abstract is classified as either being labelled with that class or not. Each classifier is trained independently and makes its prediction independently of the other classifiers. However, the fact that the classes are located in a taxonomy means that there are in fact dependencies between them; if an abstract is a positive example for *strand breaks* then it is also by definition a positive example for *genotoxic mode of action*. Such dependencies are captured by a postprocessing step in which positive classifications at a given class are propagated up the taxonomy to all higher classes.

### The CRAB tool

**Figure 6:**
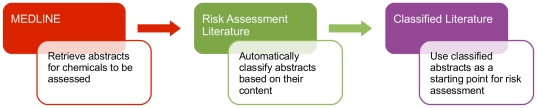
An overview of the CRAB text mining tool.

**Figure 7:**
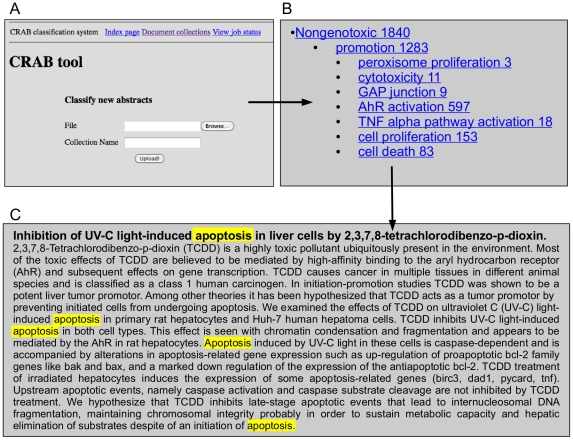
Illustration of the user interface.

In close consultation with risk assessors, we developed an online text mining tool which integrates the components described in the above sub-sections. The tool has a pipelined structure, as illustrated in Figure 6. A user can define the chemical(s) of interest and download the corresponding collection of abstracts from PubMed in XML format. The abstracts are then preprocessed and classified according to the taxonomy as described above. CRAB displays, for a given chemical, the distribution of classified abstracts over different parts of the taxonomy. The user can navigate the dataset by selecting a taxonomy class and viewing all abstracts classified as positive for that class. The user can also give feedback to the system by marking wrongly classified tags; these are then removed from display. The results are stored in a MySQL database, allowing persistent data access: the results of past sessions can be revisited and shared with other users. Figure 7 shows screenshots which illustrate some functions of the tool. We have made CRAB available to end users via an online Web interface which is accessible upon request via http://omotesando-e.cl.cam.ac.uk/CRAB/request.html.

The experiments reported here use the SVM implementation provided by the LIBSVM library [30], customised to facilitate the use of the JSD kernel. During training, we also perform feature selection to remove the many non-predictive features in the interest of enhanced efficiency and accuracy. Each feature 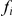 is scored according to its discriminative power over the training data using the *F-score* method of Chen and Lin [31]. Cross-validation on the training data is used to choose the proportion of features to discard; this is done by measuring performance with the top-scoring 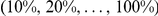 of features and keeping the subset which gives the best performance. The SVM classifier has two parameters used in training, the “cost” parameter 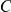 and the weight parameter 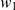 which sets the relative weighting of positive training examples; 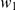 plays an important role when some labels are very rare, as in the application at hand. Similar to the feature selection process, both parameters are set through a grid search procedure that explores the range 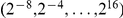.

We used a 10-fold cross-validation methodology in our evaluation: the dataset is randomly divided into 10 disjoint partitions and taking one partition at a time the classifier is trained on the other nine partitions and made to predict the labelling of the abstracts in the selected partition. In this way each abstract is labelled exactly once and we can evaluate these predictions using measures of Precision (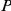), Recall (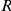) and F-measure (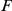, not to be confused with the F-score used for feature selection):
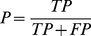
(4)

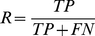
(5)

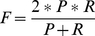
(6)where 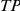, 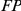 and 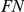 stand for the number of true positives, false positives and false negatives, respectively. These evaluation measures are standard in natural language processing and text mining. Given a set of label predictions for all data items, Precision, Recall and F-measure is computed independently for each label. In order to produce an overall performance measure these per-label scores can be averaged (*macro-average*) or single Precision and Recall figures can be calculated for the entire dataset and a *micro-average* F-measure produced using the formula in (6). Micro-averaged performance tends to be dominated by more prevalent classes, while macro-averaged performance treats all classes equally.

### User experiments and case studies

A user test was conducted to measure the acceptability of the classifier's output to risk assessors who would be using it for their work. Seven carcinogenic chemicals were selected (see the first column of Table 2); none of these chemicals had previously been used for annotation, classification or evaluation purposes. A test corpus was collected for each chemical by searching PubMed for all non-review articles mentioning the chemical that were published between 1996–2010 (as of December 7th 2010) in the journals listed in Table 3. The resulting dataset contained 2546 abstracts. As in realistic usage, many of these abstracts are irrelevant to cancer risk assessment; the classifier must distinguish relevant articles from irrelevant articles as well as assign appropriate class labels. The test corpora were submitted to the classification system for automatic annotation.

**Table 2:** User test results: total number of abstracts retrieved, number of abstracts classified as positive, Precision and interannotator agreement.

		Carcinogenic Activity			Mode of Action			Overall		
Chemical name	#	#pos	P	Agree	#pos	P	Agree	#pos	P	Agree
4-aminobiphenyl	633	94	100.0	100.0	102	97.9	97.9	128	98.6	98.6
Asbestos	571	295	99.4	98.8	183	99.6	99.8	417	99.5	99.3
Ethylene oxide	85	64	100.0	99.2	66	99.6	99.6	74	99.7	99.5
Formaldehyde	320	153	98.0	98.0	167	98.7	98.3	233	98.5	98.2
Genistein	420	127	98.7	99.6	291	99.3	99.3	341	99.3	99.4
Methylene chloride	47	25	98.7	95.5	29	100.0	100.0	34	99.3	98.6
Pyridine	470	324	98.6	99.1	317	98.7	98.6	406	98.7	98.8
Average			98.9	98.6		99.1	99.1		99.1	98.9

**Table 3:** Journals used for the user test.

Americal Journal of Industrial Medicine
Annals of Occupational Hygiene
Archives of Toxicology
Cancer Causes and Control
Cancer Detection and Prevention
Cancer Epidemiology, Biomarkers and Prevention
Cancer Letters
Cancer Research
Carcinogenesis
Chemical Research in Toxicology
Chemico-biological Interactions
DNA Repair
Environmental and Molecular Mutagenesis
Environmental Health Perspectives
Environmental Toxicology and Chemistry
European Journal of Cancer
International Journal of Cancer
International Journal of Environmental Research and Public Health
Journal of Exposure Analysis and Environmental Epidemiology
Journal of Occupational Health
Journal of Toxicology and Environmental Health A
Mutagenesis
Mutation Research
Occupational Medicine
Pathology and Oncology Research
Regulatory Toxicology and Pharmacology
The Science of the Total Environment
Toxicological Sciences
Toxicology
Toxicology and Applied Pharmacology
Toxicology Letters

The abstracts classified as positive for at least one taxonomy class were inspected by two risk assessors working independently. They decided whether the abstracts returned for each class were correctly labelled or not. After the first complete round of annotation, the level of agreement between risk assessors was calculated as the proportion of classifications about which both annotators made the same decision. We did not use the Kappa measure of interannotator agreement [32], which is often used in NLP, as it is not interpretable when the class distribution is extremely skewed: if any annotator applies the same label to all instances (in our case, carries out the desired behaviour of annotating all returned abstracts as positive) the Kappa value will be zero. The fact that the marginal distribution of classes both in the dataset itself and in the judgements of annotators affects the range of possible and probable Kappa scores has been observed in a number of studies [33]–[35]. Such studies often recommend that additional statistics be reported as an aid to better interpreting the meaningfulness of a given Kappa score; however, in the case where an annotator only uses one label the effect reaches a pathological stage where Kappa always equals zero regardless of the other annotator's decisions and there is essentially nothing to interpret.

One obvious benefit of a text mining tool such as CRAB is much improved efficiency of a major component of risk assessment: the review of existing scientific data on the chemical in question. Human risk assessors may spend months conducting partial review of relevant MEDLINE literature [16], while CRAB can perform an exhaustive review in a matter of seconds. Another major benefit is the ability to perform multi-dimensional classification of literature according to the taxonomy, i.e. the various types of scientific evidence each article offers for risk assessment. This kind of classification would be extremely difficult and time-consuming to perform by hand, especially for inexperienced risk assessors, yet it can be highly valuable because it enables both quantitative and qualitative overviews of the available data.

We conducted a number of case studies to demonstrate how such overviews can be used to support cancer risk assessment and research. The methodology of these studies involved plotting the distribution over labels assigned by the classifier to the full set of MEDLINE abstracts mentioning chemicals of direct interest to risk assessors. These quantitative findings are compared to known properties of each chemical and also used to generate new hypotheses that merit further experimental investigation.

## Results

In this section we report both direct and user-based evaluation of the classification technology, and present case studies aimed at investigating the usefulness of the CRAB tool for real life risk assessment.

### Classification results

We first took the extended taxonomy and dataset and evaluated the accuracy of the classifier directly against labels in the annotated corpus.

Figure 5 presents results for each of the 42 classes in the taxonomy with 20 or more positive abstracts; the five classes with fewer than 20 abstracts are omitted from training and testing as there is insufficient data to learn from for these very rare classes. Table 4 presents macro-averaged and micro-averaged overall results.

**Table 4:** Classification results: overall Precision, Recall and F-measure with comparison to the system of Korhonen et al. [16] on the new dataset.

	Precision	Recall	F-measure
*Overall*			
Macro-average	72.3	72.2	71.8
Micro-average	74.7	80.8	77.6
*Korhonen et al. * [*16*] * System*			
Macro-average	69.0	70.5	69.1
Micro-average	71.0	80.5	75.5

Comparing these results to those of Korhonen et al.'s [16] system on the same dataset, we find that the new system scores higher on all evaluation measures. Macro-averaged F-measure is 2.7 points higher (71.8 compared to 69.1), while micro-averaged F-measure is 2.1 points higher (77.6 compared to 75.5). Following the recommendations of Dietterich [36] we use paired 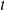-tests over the cross-validation folds to test whether this improvement is statistically significant or simply a side-effect of sampling variation; the improvement is indeed significant for both macro-averaged (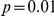, 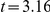, 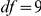, two-tailed) and micro-averaged (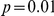, 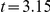) F-measure. Further investigation indicates that about half of the improvement is due to the use of the JSD kernel rather than the linear kernel and about half is due to the use of hypernyms of MeSH terms as well as the terms themselves; the use of title features has a very small positive effect. Note that the results presented here are not directly comparable to those presented earlier by Korhonen et al. [16] as our experiments use a larger taxonomy and a different, more heterogeneous (and hence more challenging) dataset; the results we use for comparison in Table 4 are new results obtained by running the old system on the new dataset and did not appear in [16].

Table 5 outlines the effect of label frequency (i.e. the number of abstracts assigned to a taxonomy class in the manually annotated dataset) on prediction accuracy. Labels which have 300 or more positive examples in the annotated dataset are easiest for the system to classify; this is not surprising, as having ¡a large number of positive examples provides the classifier with more data from which to learn a good predictive model. There is little difference between the average performance for labels with 100–299 positive examples and labels with 20–99 positive examples, suggesting that the classifier is able to predict even rare labels relatively well.

**Table 5:** Mean F-score for three frequency ranges.

Frequency range	#Labels	Average F
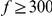	13	76.1
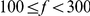	15	69.4
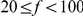	15	70.5

### User Test

The agreement figures for each chemical in the user test, measuring the proportion of retrieved abstracts for which the annotators agreed with each other, are presented in Table 2; in all cases, they are above 98%. Averaged over chemicals, agreement for the *Carcinogenic Activity* taxonomy branch is 98.6%, agreement for the *MOA* branch is 99.1% and agreement for the whole taxonomy is 98.9%. As shown by the interannotator agreement figures, the risk assessors disagreed on the correctness of some classifications. In order to produce a unanimous gold standard for calculating system precision, they revisited the cases of disagreement and settled on a reconciled decision. This allowed us to measure the precision of the system.

Precision scores for the reconciled gold standard are also presented in Table 2. The classifier's precision is very high, exceeding 99% for four chemicals and 98% for the remaining three. It was not practically feasible to perform a recall-based evaluation as well, as that would have required annotating all abstracts in the corpus with all possible labels taken into consideration.

### Case Studies

The evaluation presented in the above sections shows that the classifier is capable of assigning MEDLINE abstracts to taxonomy classes with what we consider promising accuracy (users of the system are made aware that NLP technology is never perfect and they have the ability to correct erroneous classifications). We will now investigate the practical usefulness of the tool for real-life chemical risk assessment.

**Figure 8:**
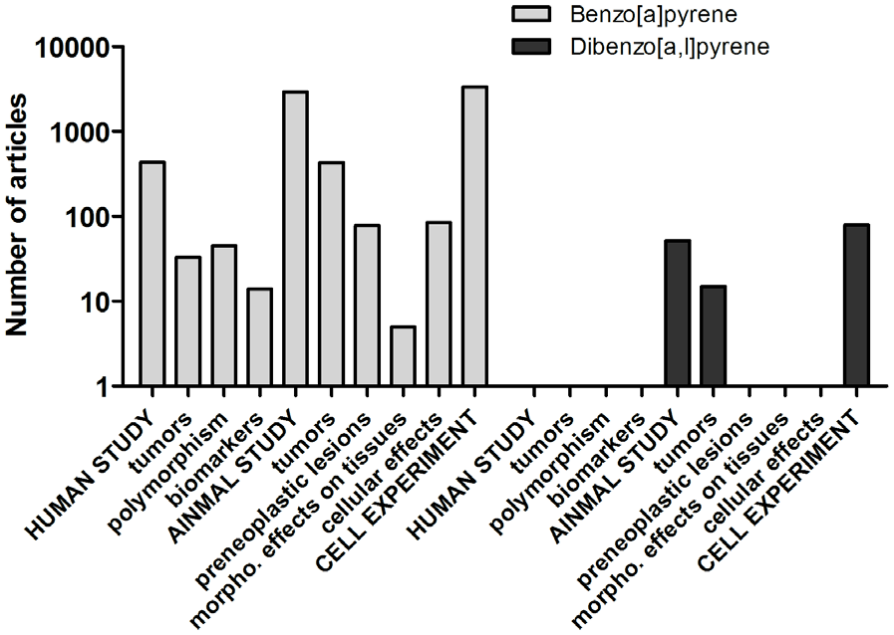
Distribution of classified abstracts over the *Scientific Evidence for Carcinogenic Activity* taxonomy for two chemicals, benzo[a]pyrene and dibenzo[al]pyrene.

First, examining the distribution of MEDLINE abstracts over the *Scientific Evidence for Carcinogenic Activity* part of the taxonomy makes it possible to see whether the key types of scientific data (animal, human and mechanistic) are already available for a chemical, or whether there are clear data gaps that need to be filled before full risk assessment can be carried out. Figure 8 shows the distribution of MEDLINE abstracts for two common chemicals, found for example as contaminants in air: benzo[a]pyrene (BP) (which had 11161 MEDLINE abstracts in total as of December 2010, 5592 assigned to the taxonomy) and dibenzo[al]pyrene (DBP) (which has 195 abstracts in total and 146 assigned to the taxonomy). It can be seen that the key types of scientific data are available for the well-studied environmental pollutant BP, while for DBP no human data is available. In this case, CRAB has revealed a serious data gap since some of the existing animal data suggest that DBP might be several orders of magnitude more carcinogenic than BP [37].

**Figure 9:**
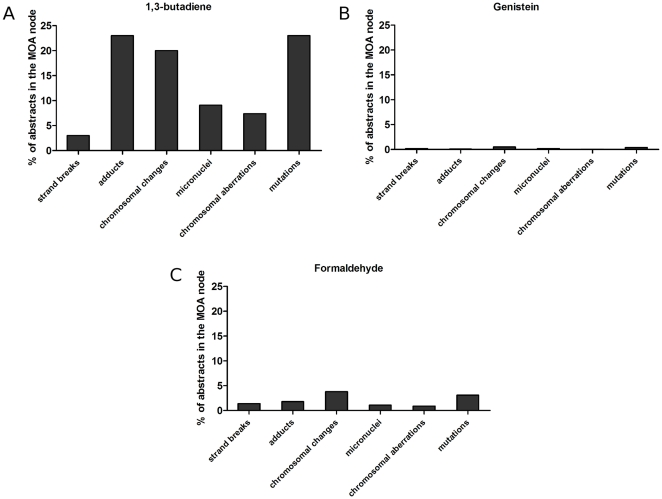
Genotoxic Mode of Action: distribution of classified abstracts for three chemicals: 1,3-butadiene, genistein and formaldehyde.

**Figure 10:**
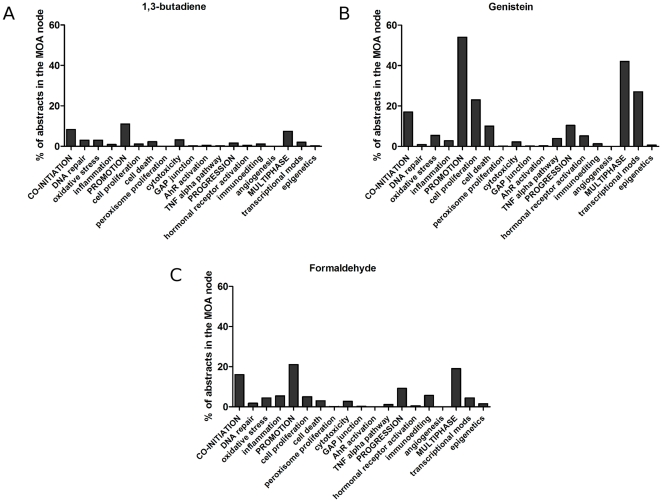
Non-genotoxic Mode of Action: distribution of classified abstracts for three chemicals: 1,3-butadiene, genistein and formaldehyde.

Secondly, for a well-researched chemical, the distribution of abstracts over the *Mode of Action* part of the taxonomy can reveal the available evidence for cancer causation as well as the likely toxicological profile of the chemical. This is illustrated in Figures 9 and 10 which show the distributions of MEDLINE abstracts for three chemicals: 1,3-butadiene, genistein and formaldehyde. Comparing the total number of MEDLINE abstracts retrieved to the number classified as relevant for MOA analysis, we see that 31.0% are retrieved for 1,3-butadiene (435 out of 1,401), 57.6% for genistein (4,908 out of 8,518) and 22.9% for formaldehyde (5,679 out of 24,757); this in itself shows how automatic analysis can dramatically cut down the reading load for a risk assessor.

1,3-butadiene is a known genotoxic chemical [38]. As expected, the clear majority (68%) of the 435 MOA abstracts include scientific data on genotoxicity (Figure 9(a)) while only 24% are classified as containing information about nongenotoxicity/indirect genotoxicity (Figure 10(a)). The latter abstracts report studies dealing with aspects of cytotoxicity, which is also expected as cytotoxicity may stimulate 1,3-butadiene-induced carcinogenesis by co-initiating or promotive effects. Figures 9(b) and 10(b) show the distribution of abstracts for genistein. It can be seen that the majority of the 4908 MOA abstracts provide scientific data on non-genotoxic effects (94%) and hormonal receptor activation (5%), which correlates to what is previously known about genistein [39]. Also shown is the profile for formaldehyde (Figures 9(c) and 10(c)). This chemical is known to induce both genotoxicity such as chromosomal changes as well as non-genotoxic effects [40]. This can be seen clearly in the distribution of 5679 abstracts over the *MOA* taxonomy, illustrating the usefulness of the tool.

**Figure 11:**
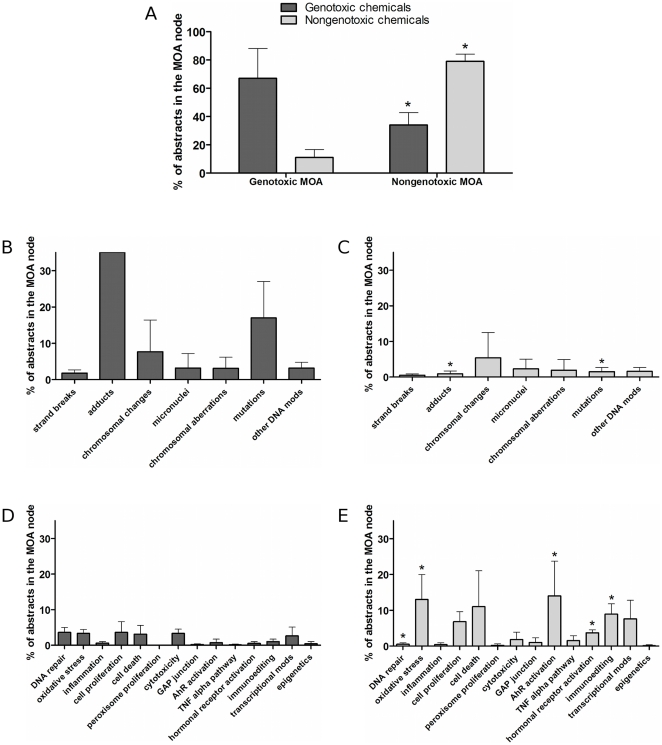
Comparison of four known genotoxic (left) and four known nongenotoxic (right) chemicals. (b–c) show the distribution in the genotoxic MOA part, (d–e) show the distribution in the nongenotoxic MOA part. The genotoxic chemicals are 1,3-butadiene, 4-aminobiphenyl, dibenzo[a,l]pyrene and ethylene oxide; the nongenotoxic chemicals are TCDD, PCB126, PCB153 and pentachlorodibenzofuran. * indicates statistically significant differences (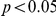, Wilcoxon rank sum test).

A similar type of analysis can be used to compare the profiles of different chemicals or chemical groups – a facility which can be particularly helpful for identifying groups of chemicals with similar toxicological profiles, or the probable group of an unknown or less researched chemical in order to get an indication of its likely properties. For example, Figure 11 shows the distribution of MEDLINE abstracts over the *MOA* part of the taxonomy for eight chemicals: TCDD, PCB126, PCB153, pentachlorodibenzofuran, 1,3-butadiene, 4-aminobiphenyl, dibenzo[al]pyrene and ethylene oxide. It reveals some striking similarities and differences between these chemicals: for example, the mean distribution of the classical tumor promoters TCDD, PCB126, PCB153 and pentachlorodibenzofuran supports the contention that these chemicals have a non-genotoxic MOA [19], [41]. In contrast, the mean distribution for 1,3-butadiene, 4-aminobiphenyl, dibenzo[al]pyrene and ethylene oxide shows a clear tendency for a genotoxic MOA [37], [38], [42], [43]. For the genotoxic group of chemicals the majority of abstracts (67
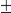
21%) were classified as genotoxic, while for the non-genotoxic group only a minority were (11
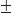
6%) (Figure 11(a)).

Similar observations can be made at the more detailed levels of the *MOA* taxonomy: the genotoxic group (Figures 11(b) and 11(c)) has a large amount of data on DNA adducts and mutations while the non-genotoxic group (Figures 11(d) and 11(e)) has more data on Ah receptor activation. As indicated above, this distribution of data corresponds to what is currently known about the MOA of these chemicals, further illustrating the accuracy and the usefulness of the tool for practical risk assessment.

**Figure 12:**
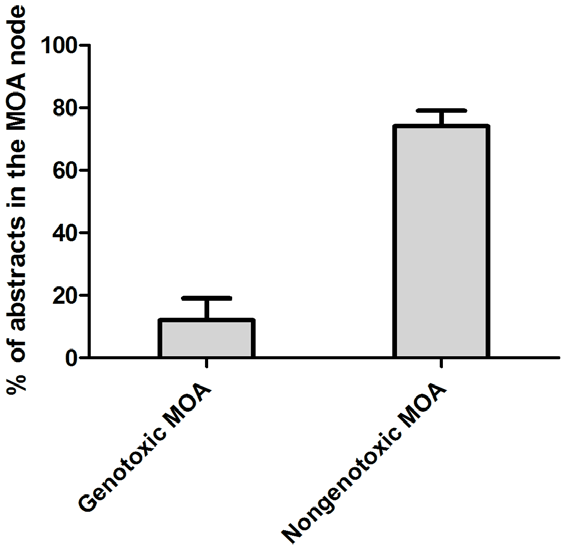
Distribution of classified abstracts over the two main MOA classes; genotoxic and nongenotoxic, for 9 antifungal chemicals used as pesticides.

**Figure 13:**
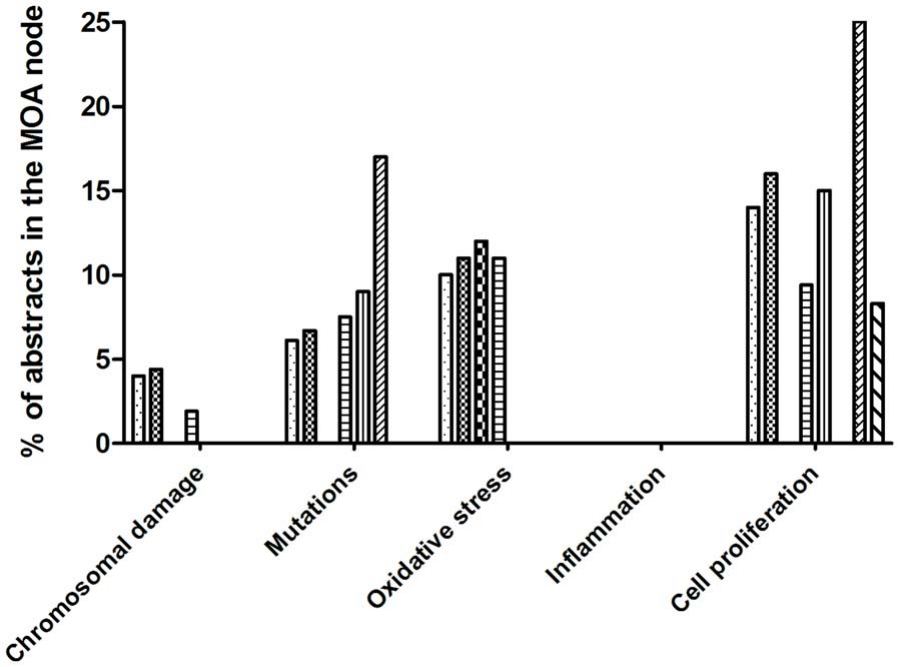
Distribution of classified triazole abstracts over some selected MOA nodes.

Next we applied the tool for a group of triazole antifungal chemicals which are used as pesticides. Humans are extensively exposed to these chemicals through e.g. consumption of food and water containing pesticide residues [44]. A concern is that this group of chemicals might have cumulative effects on human health. This calls for cumulative risk assessment, and for such an assessment it is crucial to analyse literature which describes toxicological effects that these chemicals might have in common. This is because it is likely that similar effects by two or more compounds might add up and cause cumulative effects. Figures 12 and 13 show abstracts (4–53 abstracts/chemical) dealing with 9 triazoles (cyproconazole, difenoconazole, epoxiconazole, flusilazole, muclobutanil, propiconazole, tebuconazole, triadimefon, triadimenol) distributed according to the MOA taxonomy. It can be seen that the majority (74%) of the 232 abstracts provided data on nongenotoxic effects while only 12% are classified as containing information about genotoxicity (Figure 12). Also shown is the distribution of some additional MOA nodes (Figure 13). The distribution indicates similarities between chemicals as many of the triazoles provide scientific data on cell proliferation and oxidative stress. This suggests that articles classified under these two nodes may contain information that is likely to be of interest for cumulative risk assessment of triazoles.

## Discussion

There is a need to develop text mining systems for supporting practical, literature-dependent tasks in biomedicine and to evaluate such systems not only directly, but in the context of real-life scenarios. We have introduced a new text mining tool aimed at assisting the complex task of chemical health risk assessment. The tool integrates a Web-based user interface which we have designed in collaboration with risk assessors. It enables accessing PubMed, downloading scientific abstracts on chosen chemicals, and classifying them according to multiple qualitative dimensions. The tool allows navigating the classified dataset in various ways and sharing the data with other users. We have presented direct and user-based evaluation which shows that the retrieval and classification technology integrated in the tool is highly accurate. We have also reported case studies which demonstrate how the tool can be used to support knowledge discovery in cancer risk assessment. The ability to discover novel patterns in classified data can also be useful for cancer research as it enables rapid generation of research hypotheses from published literature. These results are promising, showing that when integrated and refined in close consultation with end-users, biomedical text mining is developed enough to support fairly complex tasks in biomedicine.

From the perspective of chemical health risk assessment, the development of a text mining tool could not be timelier. There is wide-spread agreement on the need to improve the efficiency of this task. While the majority of efforts focus on the long-term future (e.g. the development of a novel system for toxicity testing), text mining can help to improve the efficiency and thoroughness of risk assessment already in the short to medium term future. Our tool is aimed at assisting the first, time-consuming component of risk assessment which is currently conducted largely manually: the gathering and analysis of existing scientific data on the chemical in question. For risk assessment under real-world conditions, the retrieved and classified full articles will need to be examined in detail by risk assessors. CRAB can support this process in several ways. Since it classifies scientific literature according to the type, amount and strength of the evidence it provides for risk assessment, it can help assessors focus on articles which are likely to be the most relevant starting points. Individual articles can be opened easily and the different types of scientific data they contain can be highlighted, supporting effective review of the scientific literature.

CRAB can be developed further in various ways. The taxonomy can be extended to cover other types of health risks (e.g. allergy, endocrine disruption, among many others) with a minimum of effort: users of the tool can create a new sub-taxonomy for a specific health risk when required and effectively develop and extend the sub-taxonomy while using the tool for their work. After re-training the classifier accordingly, the system can be be used to support other important areas of chemical health risk assessment.

In addition, the tool could be improved in other ways. It could be modified to distinguish between positive and negative evidence for a particular risk or to distinguish between reported fact and speculation. Risk assessment of groups of chemicals with similar toxicological profiles is often discussed as a means to speed up the process; the CRAB tool may facilitate the selection of chemicals to be included in such groups and the selection of chemicals that may have common effects of interest for cumulative risk assessment. The literature search functionality can be extended to access other relevant literature databases. The classification can be refined to consider journal impact factors, citation frequencies, and cross references, helping risk assessors to identify e.g. more prominent, less important and incremental published studies, as well as studies forming clusters. The tool can also be extended to support analysis of the scientific data and the subsequent writing of risk assessment reports.

Clearly, further development is required before a fully ideal tool designed to support literature gathering and analysis in chemical risk assessment at large is available “off the shelf”. However, the tool and research we have presented in this paper illustrate the many ways in which text mining could help to improve the efficiency and quality of chemical risk assessment, as well as free risk assessors to focus on what they are best at: expert judgement.

## Supporting Information

Information S1 **Index of the label names used in the annotated dataset.**
(TXT)

Information S2 **Annotated dataset.**
(TXT)
